# Urban-Rural Comparison of Knowledge and Practices of Diabetes Mellitus Among Type 2 Diabetic Population in Telangana: A Cross-Sectional Study

**DOI:** 10.7759/cureus.92626

**Published:** 2025-09-18

**Authors:** Garima Misra, Triven Sagar Sandepogu, Vikas Saharan, Kirti K Hinduja, Stefi M S

**Affiliations:** 1 Neurology, ESIC Medical College and Hospital, Hyderabad, IND; 2 Internal Medicine, ESIC Medical College and Hospital, Hyderabad, IND; 3 Internal Medicine, Northern Railways Divisional Hospital, Ambala Cantonment, IND; 4 General Medicine, Urban Primary Health Center, Kalyan, IND; 5 Community Medicine, ESIC Medical College and Hospital, Hyderabad, IND

**Keywords:** a cross-sectional study, diabetes mellitus type 2, knowledge, practices, rural population, urban population

## Abstract

Background: Diabetes mellitus has become a highly common noncommunicable condition. An in-depth understanding of the disease condition, its influencing factors, and effective management practices can provide numerous benefits to the diabetic population. Not only does it prevent the complications associated with diabetes, but it also helps people make informed decisions regarding the management choices and ensures better compliance. The study aims to determine the knowledge and practices regarding diabetes mellitus among the type 2 diabetic population.

Methods: The study design was cross-sectional, involving a total of 200 participants who attended the outpatient department (OPD) of the urban and rural field practice areas under ESIC Hospital, Hyderabad, Telangana. The study was conducted over two months. A semistructured, interviewer-administered questionnaire was prepared in the patient's preferred languages to evaluate the knowledge and practices of diabetes mellitus among the type 2 diabetic population. Statistical analysis was performed using IBM SPSS Statistics for Windows, Version 21 (Released 2012; IBM Corp., Armonk, New York, United States), with the statistical significance fixed at p-values less than 0.05.

Results: Our study found that the average age of the study population was 56.72, and most of them were males. The urban participants outnumbered the rural participants. The data obtained were compared between the urban and rural settings. We observed a statistically significant (p < 0.0001) relationship in the educational status of diabetic patients in the urban versus rural areas. The proportion of educated participants was higher in the urban areas. Similarly, the illiteracy rate was found in 10 (18.86%) and eight (5.44%) rural and urban participants, respectively, showing a higher proportion in the rural setting. In addition to this, the results revealed a significant (p = 0.0214) link between the perceived risk factors of diabetes, with 124 of the 470 (26.38%) responses given by the participants citing a stressful lifestyle as the major contributor. Among the most common organs affected by diabetes, the kidneys emerged as the most common response. Furthermore, the most popular management strategies adopted for diabetes were exercise by 103 (32.08%), diet by 101 (31.46%), and medications by 97 (30.21%) participants, with the urban and rural populations relying more on medicines and diet plus exercise, respectively. However, this was not statistically significant.

Conclusion: The study thus successfully narrates the current level of awareness about type 2 diabetes in Telangana. It highlights some valid differences in the data collected from the urban and rural participants as well. This adds to the existing knowledge database, which is already limited, and facilitates further policy development.

## Introduction

Diabetes mellitus has emerged as one of the rapidly increasing public health concerns in this generation. It is not only a chronic disease, but a precursor to life-altering complications like stroke, blindness, cardiovascular disease, kidney failure, and neuropathy [1]. The International Diabetes Federation (IDF) reports that 537 million people were living with diabetes in 2021, and this is expected to rise to 783 million by 2045 [2]. According to the World Health Organization (WHO), the prevalence of diabetes is increasing most quickly in low- and middle-income countries [3]. This can be attributed to poor health-related knowledge, limited availability of health services, and inadequate resources for early diagnosis and consistent treatment. In India, 69.2 million people were living with diabetes in 2015, with this number expected to reach 98 million by 2030 [4]. Effective diabetes management requires regular exercise, a healthy diet, being consistent with medications, and regular follow-up appointments, hence being heavily dependent on the patient's behavior and daily practices. Early detection and prevention rely on awareness of risk factors and prevention. As far as we know, there is a lack of data about knowledge and awareness of diabetes among the general population of Telangana state, India [4]. Without understanding what people know, believe, and do about diabetes, health strategies risk being ineffective or misdirected. This study aims to understand the knowledge and practice regarding diabetes in people of the Telangana state, India. This research helps fill a void in the local data and adds to the existing knowledge base of the patients, providing a strong foundation for devising policies and programs on health education.

## Materials and methods

This cross-sectional study was conducted at the outpatient department (OPD) of the urban and rural field practice areas under ESIC College and Hospital, Hyderabad, over two months, from February to April 2025. The Institutional Ethics Committee (IEC) has reviewed the project bearing no. ESICMC/SNR/IEC-S0491/01-2025 version no. V01 and has unanimously approved the proposed project. The study population included diagnosed type 2 diabetes mellitus patients, aged 19 years or above, who provided consent. The exclusion criteria included pregnant females and patients who refused to provide consent or denied participating further in the study. A study by Mathur et al. on the prevalence, awareness, treatment, and control of diabetes in India was used as the reference for calculating sample size, where the prevalence of awareness of diabetes mellitus was (p) 0.458 [5]. The complement of this proportion (q) was 0.542, and a margin of error (e) of 0.07 was used. Using the standard formula for sample size calculation in cross-sectional studies, $n = \frac{Z^{2} \cdot p \cdot q}{e^{2}}$, where the Z (Z = 1.96) represents the standard normal variate for a 95% confidence interval (1.96), the minimum sample size required was 194. However, to account for nonresponses, 200 participants were ultimately enrolled, comprising 147 urban and 53 rural residents. Convenient sampling was used as participants presented to OPDs at the urban and rural field practice areas under ESIC College and Hospital, Hyderabad, until the desired size of the sample was achieved.

A semistructured, interviewer-administered questionnaire (in the appendices) was used to gather data. The questionnaire was translated from English to Telugu and Hindi, depending on the participant’s preferred language. Interviews were conducted in a private setting to ensure confidentiality. It was divided into two parts: sociodemographic characteristics and assessment of diabetes knowledge and management practices of the participants. The data were analyzed using MS Excel (Microsoft Corporation, Redmond, Washington, United States) and GraphPad Prism software version 5 (GraphPad Software, Inc., San Diego, CA). Data for continuous variables is expressed as mean ± SD, and percentages for categorical data. The comparison between the two groups was done by the Chi-square test/Fisher’s exact test for categorical data. All p-values less than 0.05 were deemed statistically significant.

## Results

In our study, a total of 200 consenting participants from urban and rural areas were surveyed. Out of these, 147 belonged to urban areas and 53 belonged to rural areas (depending upon how many diabetic patients were received during sample collection from each area). The maximum number of participants belonged to the 49-58-year age group, with a mean age of 56.72 years. A total of 114 (57%) of the participants surveyed were males, with a higher proportion in urban areas. A statistically significant difference (p < 0.0001) was noted in the education among the urban and rural participants, with 53 participants (26.50%) having completed middle school as their highest level of education. Among these, the urban participants accounted for 41 (76.37%), notably exceeding the proportion among rural residents. Illiteracy was 13.42% more prevalent in rural settings than in urban areas. Nearly half of the respondents in the study had type 2 diabetes mellitus for the last 0-5 years. The sociodemographic characteristics of the participants, classified based on their residence, are shown in Table 1. This is followed by Table 2, which shows the understanding of the diabetes risk factors and the organ impact among urban and rural participants. 

**Table 1:** Sociodemographic characteristics of the study participants based on their residence

Sociodemographic features	Residence		
	Urban n (%), N = 147	Rural n (%), N = 53	Total n (%)
Age			
19-28	3 (2.04)	0 (0)	3 (1.5)
29-38	4 (2.72)	2 (3.77)	6 (3)
39-48	23 (15.64)	8 (15.09)	31 (15.5)
49-58	54 (36.73)	27 (50.94)	81 (40.5)
59-68	37 (25.17)	11 (20.75)	48 (24)
69-78	20 (13.60)	4 (7.54)	24 (12)
79-88	6 (4.08)	1 (1.88)	7 (3.5)
Gender			
Male	89 (60.54)	25 (47.16)	114 (57)
Female	58 (39.45)	28 (52.83)	86 (43)
Educational qualifications			
Illiterate	8 (5.44)	10 (18.86)	18 (9)
Primary school	8 (5.44)	9 (19.68)	17 (8.5)
Middle school	34 (23.12)	19 (35.84)	53 (26.5)
High school	41 (27.89)	9 (16.98)	50 (25)
Graduate	43 (29.25)	5 (9.43)	48 (24)
Postgraduate	13 (8.84)	1 (1.88)	14 (7)
Years living with diabetes			
0-5 years	63 (43.44)	29 (52.72)	92 (46)
5-10 years	42 (28.96)	15 (27.27)	57 (28.5)
10-15 years	26 (17.93)	7 (12.72)	33 (16.5)
15-20 years	5 (3.44)	1 (1.81)	6 (3)
More than 20 years	9 (6.20)	3 (5.45)	12 (6)

**Table 2:** Knowledge of diabetes risk factors and the organs affected among urban and rural study participants The study participants gave multiple responses for the same question asked in this section. Hence, n refers to the number of responses rather than the number of participants. Percentages were calculated using the total number of responses as the denominator

Knowledge	Residence		
	Urban n (%), N = 343 responses	Rural n (%), N = 127 responses	p-value
Risk factors of diabetes			
Raised blood pressure	69 (20.11)	11 (8.66)	0.0214
Overweight	65 (18.95)	23 (18.11)	
Family history	48 (13.99)	24 (18.89)	
Stressful lifestyle	86 (25.07)	38 (29.92)	
Sedentary lifestyle	63 (18.36)	30 (23.62)	
Don't know	12 (3.49)	1 (0.78)	
Organs affected by diabetes	Urban n (%), N = 348 responses	Rural n (%), N = 126 responses.	p-value
Kidney	80 (22.98)	36 (28.57)	0.7199
Eye	56 (16.09)	21 (16.66)	
Heart	55 (15.80)	22 (17.46)	
Nerves	30 (8.62)	8 (6.34)	
Feet	42 (12.06)	17 (13.49)	
Lungs	21 (6.03)	6 (4.76)	
Brain	34 (9.77)	7 (5.55)	
Don't know	30 (8.62)	9 (7.14)	

Among the questions asked to assess the knowledge of the study participants about diabetes, a statistically significant difference (p = 0.0214) was noted among the perceived risk factors between the urban and rural populations. Overall, 53 (26.38%) cited a stressful lifestyle as the most common risk factor for diabetes mellitus. The urban participants considered raised blood pressure as a risk factor, which is 11.45% more than the rural population. Additionally, kidneys were the most frequently mentioned organ affected by diabetes, accounting for 49 (24.47%) of the responses.

The most frequently reported diabetes management strategies were exercise (64, 32.08%), diet modification (63, 31.46%), and medication use (60, 30.21%). Urban participants reported more reliance on medications as compared to the rural population, which relied more on diet and exercise. However, the results about the management practices were statistically insignificant. More than half of the participants had not practiced foot care or had a foot examination done in the last year, which was comparable in urban and rural respondents. Similarly, almost half of the participants reported having an eye checkup done only when their vision was affected. Table 3 represents the management practices of the diabetic patients in the urban and rural settings. 

**Table 3:** Management practices among the diabetic population in urban and rural participants in the study The study participants gave multiple responses for the same question about management practices; however, single responses were given when asked about foot or eye checkups

Practices	Residence		
	Urban n (%), N = 231 responses	Rural n (%), N = 90 responses	p-value
Approach to management			
Diet	70 (30.30)	31 (34.44)	0.5
Medications	74 (29.93)	23 (25.55)	
Exercise	71 (30.73)	32 (35.55)	
Didn’t use any	16 (6.92)	4 (4.44)	
Foot examination in the last year	Urban n (%), N = 147 responses	Rural n (%), N = 53 responses	p-value
Yes	44 (29.93)	16 (30.18)	0.33
No	92 (62.58)	36 (67.92)	
Can’t recall	11 (7.48)	1 (1.88)	
Frequency of eye checkups	Urban n (%), N = 147 responses	Rural n (%), N = 53 responses	p-value
Six monthly	29 (19.72)	8 (15.09)	0.52
Only when vision is affected	69 (46.93)	21 (39.62)	
Yearly	18 (12.24)	10 (18.86)	
Two yearly	4 (2.72)	3 (5.66)	
Didn’t attend any	27 (18.36)	11 (20.75)	

## Discussion

In the current study, several statistically significant differences were found between urban and rural populations. Notably, the educational status was higher among urban participants (p < 0.0001). Additionally, the urban participants demonstrated better knowledge of risk factors (p = 0.0214), with a stressful lifestyle being the most common risk factor cited across both groups. When considering the impact of diabetes, the participants most frequently mentioned the kidneys as the most affected. Lastly, performing exercise (64, 32.08%) was the most common approach to management mentioned by the participants, followed by dietary changes (63, 31.46%) and medications (60, 30.21%). 

Most participants are in the 49-58 age group, with a mean age of 56.72 years. A total of 114 (57%) of the participants are males, more so in the urban population. Comparable findings were observed in a study by Dahake et al. with mean ages of 50.45 (urban) and 52.50 (rural) years [6]. Men appear to be more vulnerable to the effects of physical inactivity and obesity, likely due to variations in insulin sensitivity and fat distribution patterns, and thus develop type 2 diabetes at a reduced body mass index, which may explain their increased susceptibility [7]. Illiteracy was 13.42% higher in the rural population. More urban participants had completed higher and secondary education. But education alone does not necessarily guarantee increased compliance with the treatment and control measures. A South African study showed that despite 68% and 64.5% of participants having good knowledge and positive attitudes, only 35.8% consistently followed appropriate practices, highlighting the role of behavior, affordability, and habit formation in determining diabetes outcomes [8]. 

A total of 53 (26%) study participants perceived that a stressful lifestyle contributes substantially as a risk factor for diabetes mellitus. In a similar study aimed at evaluating the knowledge of the diabetic population in Trivandrum by Sathish et al., family history was the most perceived risk factor among the people surveyed [9]. This can be because most of our participants are middle school-educated factory workers facing intense urban pressures in Hyderabad; they may be more likely to view daily psychosocial and financial stresses as the main drivers of diabetes risk. In our study, the kidneys (49, 24.47%) were the most mentioned organ affected by diabetes, with nerves, heart, and lungs being the least mentioned organs. Similar findings were mentioned by the Indian Council of Medical Research (ICMR) study [10]. 

In the urban cohort, almost 30% of the population relied on diet, exercise, and medications each, whereas in the rural population, almost 35% of the population relied on diet and exercise each, and only 25% relied on medications. However, in a study by Goyal et al., adherence to medications, exercise, and diet was better than in contrast to our study [11]. This difference could be due to the variation in cohorts; our participants were mostly newly diagnosed and dependent on periodic visits to the field practice area, while those in the Goyal et al. study likely had better access to regular follow-ups and structured counselling through noncommunicable disease (NCD) clinics, which may have supported stronger habit formation and adherence to treatment strategies. However, the relatively modest rates of adherence across all strategies in our study also point to the need for behavioral reinforcement and structured diabetes education, regardless of geography.

Awareness regarding the effects of diabetes on the eyes and feet is limited in most participants. Only 60 (30%) participants had a foot exam in the last year. These findings are in line with the studies by Goyal et al. and Gowthaman et al., which reported adequate foot care in only 41.6% and 33.3% of patients, respectively [11,12]. Nearly 90 (45%) participants underwent eye checkups only when their vision was affected, rather than on a routine basis. Similarly, Achar et al. reported that only 33 (40.2%) participants underwent regular eye examinations [13]. This suggests a lack of awareness regarding the effect of diabetes on different organ systems, implying the need for better educational strategies to raise awareness. In addition to this, educating diabetic patients about self-care management practices has become an important aspect of improving health outcomes [14].

This study had certain limitations. We did not explore the participants' attitude about diabetes management, which may be the missing link in keeping knowledge from becoming action. Moreover, the responses regarding knowledge and practices were self-reported and may be subject to social desirability bias, especially in questions related to lifestyle practices and adherence. Additionally, the questionnaire used in the study, although not formally validated in our population, was adapted from previous studies [15]. The sample had unequal subgroup sizes (147 urban and 53 rural). As the study was primarily descriptive, this imbalance did not affect the overall estimates, but some subgroup analyses could be underpowered. Hence, the future studies can plan to have prospective study designs to allow stronger analytical conclusions.

## Conclusions

This cross-sectional study highlights a significant knowledge-practice gap in diabetes management, both at an urban and rural level. Despite having knowledge about diabetes, such as risk factors and organs affected, the practical aspects, like regular foot and eye checkups, were inadequately practiced. These findings underscore the need for targeted interventions that will not only enhance knowledge but will also address the barriers to bridging the gap between knowledge and practice. In addition to this, the public health programs should also prioritize access to healthcare in both urban and rural settings, to translate knowledge into practice and ultimately improve patient outcomes.

## Appendices

**Table 4:** Questionnaire used for the study

Questions	Options/answer
Informed consent	Yes
	No
Age	
Gender	Male
	Female
Residence	Urban
	Rural
Educational qualifications	Illiterate
	Primary school
	Middle school
	High school
	Graduate
	Postgraduate
For how long have you had type 2 diabetes mellitus	0-5 years
	5-10 years
	10-15 years
	15-20 years
	More than 20 years
Which of the following factors do you feel pose a risk for developing type 2 diabetes mellitus	Raised blood pressure
	Overweight
	Family history
	Stressful lifestyle
	Sedentary lifestyle
	Don't know
Which of the organs do you feel are affected due to type 2 diabetes mellitus?	Kidney
	Eye
	Heart
	Nerves
	Feet
	Lungs
	Brain
	Don't know
What is your approach towards managing type 2 diabetes mellitus	Diet
	Medications
	Didn't use any
Foot examination in the last year	Yes
	No
	Didn't attend any
Frequency of eye checkups	6 monthly
	Only when vision is affected
	Yearly
	2 yearly
	Didn't attend any
